# Supplementary material for: Awareness of diagnosis, treatment plan and prognosis among patients attending public hospitals and health centers in Addis Ababa, Ethiopia
Source: PLoS One. 2022 Jun 23;17(6):e0270397. doi: 10.1371/journal.pone.0270397 (PMC9225456; doi:10.1371/journal.pone.0270397)
Supplement: Attachment — Submitted filename: Response to Reviewers.docx [file pone.0270397.s001.docx]

Date Jun 5, 2021

Kingston Rajiah
Academic Editor
PLOS ONE

**Re: Awareness of diagnosis, treatment plan and prognosis among patients attending public hospitals and health centers in Addis Ababa, Ethiopia.**


Dear Dr. Kingston Rajiah,

Thank you for review of our manuscript. We found your comments and reviewers’ comments very helpful. We have revised the manuscript based on your comments. We have attached:

1. **Response to Reviewers** - A rebuttal letter that responds to each point raised by the academic editor and reviewer(s).
2. **Revised Manuscript with Track Changes** - A marked-up copy of your manuscript that highlights changes made to the original version.
3. **Manuscript** - An unmarked version of your revised paper without tracked changes.

We look forward to receiving hearing from you.

Kind regards,

Dr Alula M Teklu

On behalf of the authors

**Responses to Editor’s comments**
1. Please ensure that your manuscript meets **PLOS ONE's style requirements**, including those for file naming. The PLOS ONE style templates can be found at

<https://journals.plos.org/plosone/s/file?id=wjVg/PLOSOne_formatting_sample_main_body.pdf> and

<https://journals.plos.org/plosone/s/file?id=ba62/PLOSOne_formatting_sample_title_authors_affiliations.pdf>

**Response**: We have edited the title page and file names based on the PLOS ONE’s style requirements.

2. We suggest you thoroughly **copyedit your manuscript for language usage**, spelling, and grammar. If you do not know anyone who can help you do this, you may wish to consider employing a professional scientific editing service.  

Whilst you may use any professional scientific editing service of your choice, PLOS has partnered with both American Journal Experts (AJE) and Editage to provide discounted services to PLOS authors. Both organizations have experience helping authors meet PLOS guidelines and can provide language editing, translation, manuscript formatting, and figure formatting to ensure your manuscript meets our submission guidelines. To take advantage of our partnership with AJE, visit the AJE website (http://learn.aje.com/plos/) for a 15% discount off AJE services. To take advantage of our partnership with Editage, visit the Editage website ([www.editage.com](http://www.editage.com/)) and enter referral code PLOSEDIT for a 15% discount off Editage services.  If the PLOS editorial team finds any language issues in text that either AJE or Editage has edited, the service provider will re-edit the text for free.

***Response****: The manuscript has now been copyedited for language usage by Christopher Crompton who is an experienced editor.*

Upon resubmission, please provide the following:

- The name of the colleague or the details of the professional service that edited your manuscript
- A copy of your manuscript showing your changes by either highlighting them or using track changes (uploaded as a *supporting information* file)
- A clean copy of the edited manuscript (uploaded as the new *manuscript* file)

***Response****: We have included these files as per the PLOS ONE guidelines*

3. Please include additional information regarding the survey or questionnaire used in the study and ensure that you have provided sufficient details that others could replicate the analyses. For instance, if you **developed a questionnaire** as part of this study and it is not under a copyright more restrictive than CC-BY, please include a copy, in both the original language and English, as Supporting Information, or include a citation if it has been published previously.

***Response****: English and Amharic version of the questionnaire and the consent forms that we developed as part of this study are attached as supplemental file.*

4. In the Methods, please discuss whether and how the questionnaire was validated for your context and/or pre-tested. If these did not occur, please provide the rationale for not doing so.

***Response****:*  *After training of the data collectors, the data collection process and questionnaire were pre-tested in similar contexts. Refinements to the data collection process and questionnaire were made based on the lessons from the pre-test. This information is included in the revised version*

5. In your statistical analyses, please state whether you accounted for clustering by locality. For example, did you consider using multilevel models?

***Response****: In the analysis of factors associated with awareness of diagnosis, treatment and prognosis, we controlled for the effects of potential confounders including type of facilities and departments within facilities. As the sample is proportionally distributed between hospitals and health centers, we don’t expect significant effect of clustering.*

6. In statistical methods, please refer to any **post-hoc** corrections to correct for multiple comparisons during your statistical analyses. If these were not performed please justify the reasons. Please refer to our statistical reporting guidelines for assistance (<https://journals.plos.org/plosone/s/submission-guidelines.#loc-statistical-reporting>).

***Response****: Most of our variables are categorical. Post-hoc multiple comparisons were not needed for these types of analysis. In the logistic regression models, we compared each category against the reference category.*

7. Thank you for stating the following in the Financial Disclosure section:

[This study was funded by MERQ PLC.].

We note that one or more of the authors have an affiliation to the commercial funders of this research study: MERQ consultancy PLC

1. Please provide an amended Funding Statement declaring this commercial affiliation, as well as a statement regarding the Role of Funders in your study. If the funding organization did not play a role in the study design, data collection and analysis, decision to publish, or preparation of the manuscript and only provided financial support in the form of authors' salaries and/or research materials, please review your statements relating to the author contributions, and ensure you have specifically and accurately indicated the role(s) that these authors had in your study. You can update author roles in the Author Contributions section of the online submission form.

***Response****: The authors’ contribution section is amended as suggested.*

 Please also include the following statement within your amended Funding Statement.

“The funder provided support in the form of salaries for authors [insert relevant initials], but did not have any additional role in the study design, data collection and analysis, decision to publish, or preparation of the manuscript. The specific roles of these authors are articulated in the ‘author contributions’ section.”

If your commercial affiliation did play a role in your study, please state and explain this role within your updated Funding Statement.

***Response****: The funding statement is amended as suggested.*

2. Please also provide an updated Competing Interests Statement declaring this commercial affiliation along with any other relevant declarations relating to employment, consultancy, patents, products in development, or marketed products, etc.

Within your Competing Interests Statement, please confirm that this commercial affiliation does not alter your adherence to all PLOS ONE policies on sharing data and materials by including the following statement: "This does not alter our adherence to  PLOS ONE policies on sharing data and materials.” (as detailed online in our guide for authors http://journals.plos.org/plosone/s/competing-interests). If this adherence statement is not accurate and  there are restrictions on sharing of data and/or materials, please state these. Please note that we cannot proceed with consideration of your article until this information has been declared.

***Response****: The competing interests statement is amended.*

Please include both an updated Funding Statement and Competing Interests Statement in your cover letter. We will change the online submission form on your behalf.

Please know it is PLOS ONE policy for corresponding authors to declare, on behalf of all authors, all potential competing interests for the purposes of transparency. PLOS defines a competing interest as anything that interferes with, or could reasonably be perceived as interfering with, the full and objective presentation, peer review, editorial decision-making, or publication of research or non-research articles submitted to one of the journals. Competing interests can be financial or non-financial, professional, or personal. Competing interests can arise in relationship to an organization or another person. Please follow this link to our website for more details on competing interests: http://journals.plos.org/plosone/s/competing-interests

***Response****: Updated Funding Statement and Competing Interests Statement are included in the updated cover letter*

8. We note that you have indicated that data from this study are available upon request. PLOS only allows data to be available upon request if there are legal or ethical restrictions on sharing data publicly. For information on unacceptable data access restrictions, please see <http://journals.plos.org/plosone/s/data-availability#loc-unacceptable-data-access-restrictions>.

In your revised cover letter, please address the following prompts:

a) If there are ethical or legal restrictions on sharing a de-identified data set, please explain them in detail (e.g., data contain potentially identifying or sensitive patient information) and who has imposed them (e.g., an ethics committee). Please also provide contact information for a data access committee, ethics committee, or other institutional body to which data requests may be sent.

b) If there are no restrictions, please upload the minimal anonymized data set necessary to replicate your study findings as either Supporting Information files or to a stable, public repository and provide us with the relevant URLs, DOIs, or accession numbers. Please see <http://www.bmj.com/content/340/bmj.c181.long> for guidelines on how to de-identify and prepare clinical data for publication. For a list of acceptable repositories, please see <http://journals.plos.org/plosone/s/data-availability#loc-recommended-repositories>.

 We will update your Data Availability statement on your behalf to reflect the information you provide.

***Response****: Data contain potentially identifying patient information. Requests for access to data can be submitted to the corresponding author who can process data access.*

9. We note you have included a table to which you do not refer in the text of your manuscript. Please ensure that you refer to Table 2 in your text; if accepted, production will need this reference to link the reader to the Table.

***Response****: Included.*

10. Please include your tables as part of your main manuscript and remove the individual files. Please note that supplementary tables (should remain/ be uploaded) as separate "supporting information" files.

***Response****: All tables are within the main manuscript*

**Reviewers' comments:**

**Comments to the Author**

Reviewer #1:

1. An overall comment is for the quality of writing: English language is sometimes not the most appropriate. I urge authors to check the text thoroughly before resubmission and to read other published papers from the journal to check the style of writing.

***Response****: The language has been carefully edited by Christopher Crompton.*

2. The author must add the inclusion and exclusion criteria in the study.

***Response****: A sub-section on inclusion/exclusion criteria is included in the revised version.*

3. The research work Awareness of diagnosis, treatment plan and prognosis among patients attending public hospitals and health centers in Addis Ababa, Ethiopia is well conducted among the patients, the sample size is to be increased, and further work may be carried out.

***Response****: We agree with this suggestion and included it in our recommendations section*

4. The authors also add the limitations of the study.

***Response****: Study limitations are described in the last paragraph of the discussion section*

Reviewer #2:

General comment: this study examines an important question about patients perception and understanding of care received. Inconsistencies in data and need of further clarity on methodology need to be examined.

***Response****: We have revised the manuscript to improve clarity and ensure consistency.*

Specifics:
Abstract: “Patients with good experience at their clinical assessment had significantly lower awareness of their prognosis"
Conclusion: “Patients with good experience at their clinical assessment had significantly higher awareness of their prognosis.”

--> both statements are in contradiction, also "(OR=0.25; 95% CI: 0.08, 0.81)” in the abstract does not correspond to the data in the table

**Response**: We have revised the text in the conclusion section and verified the data presented in the abstract against table 3.

Did the authors collect data on gender of health care providers? Was there a difference in patient knowledge of diagnosis, treatment plan or prognosis depending on provider gender? If provider gender was not examined would perhaps rephrase: “Patient care is shifting globally towards patient-centered approach [1, 2].”

**Response**: Data on gender of the provider was not collected. We have revised the text accordingly.

“Questionnaire (PEQ) (25), Patient Perception of Patient-Centeredness (PPPC) scale (26), Perception on Quality of Health Service, General Patient Satisfaction Scale (27) and Items on Patients Awareness on their Diagnosis, treatment and Prognosis (28).”

was this translated or did the interviewer use the English version and each translate themselves? Were these instruments validated in the translated language? What language was used? Please provide a copy of the English and translated version as an appendix. Some validation of the instruments would be useful for this and future publications. This will also help readers to better understand how variables such as "patient centeredness" or "prognosis" or the various satisfaction/good care variables were defined.

***Response****: The English version of the tool was translated to Amharic by expert translators. The translated version was reviewed by experts and was the pre-tested before use. We have provided a copy of both the English and Amharic versions of the questionnaire.*

Also authors could provide a reference indicating that documenting prognosis separate from the diagnosis and treatment plan is a requirement in medical notes. Is this now a requirement in Ethiopia, WHO, etc for example? Often prognosis is implicit in the diagnosis and treatment plan in medical notes.

***Response****: Yes, information about prognosis is also implicit in Ethiopia. We asked the patient if he or she knows whether his/her diagnosis is curable or not. For most of the information, we asked the treating physician about prognosis. Overall, prognostic information was largely inferred from the diagnosis.*

“We used logistic regression models to assess factors associated with awareness of diagnosis, treatment plan and prognosis”
What variables were included in these models? Has were interactions assessed and managed? It is not clear in the results section which results were bivariate, which were from multivariate models and or variables were included in the models.

***Response****: All the variables in table 3 were included in each logistic regression model (awareness of diagnosis, treatment and prognosis). We presented only adjusted ORs. We presented descriptive stats (bivariate) in table 2. In the revised version, we have indicated the variables and included in the models and that the ORs are adjusted.*

Gynecologic patients had significantly lower awareness about their diagnosis and treatment plan, compared to who? Why is this different from females in general?

***Response****: This is compared to the reference category (patients in general outpatient department). We have controlled in the effect of gender in the model. But the reason behind this needs further study.*

How could patients understand prognosis if they don’t know the diagnosis or treatment plan?

***Response****: The question we asked about prognosis is generic (whether the condition is treatable or not) and doesn’t need specific knowledge of diagnosis or details of treatment plan.*

Also in the introduction would indicate the location of the references like in the discussion.

***Response****: We used the same reference style in both the introduction and discussion section.*

% in the text of the result would be better in a table.

**Response**: We have reduced some of the %s indicated in the text.

Data from the tables to keep tract
Awareness of Diagnosis Awareness of Treatment Awareness of Prognosis
Female
179(63.3) 163(54.2) 244(72)
1.40(0.91,2.15) 0.129 1.20(0.8,1.81) 0.374 1.17(0.73,1.86)0.511

Gynecology
31(48.4) 29(43.9) 64(77.1)
0.29(0.12,0.73) 0.009 0.30(0.12,0.77) 0.013 0.92(0.34,2.52) 0.879

General satisfaction (Ref: Not satisfied)
131(58.5) 116(48.5) 166(61.5)
0.68(0.43,1.05) 0.082 0.75(0.49,1.14) 0.179 0.75(0.47,1.19) 0.222

***Response****: These data from Table 2 and 3 go together. However, to keep the tables simpler, we put them in two tables.*

6. PLOS authors have the option to publish the peer review history of their article ([what does this mean?](https://journals.plos.org/plosone/s/editorial-and-peer-review-process#loc-peer-review-history)). If published, this will include your full peer review and any attached files.


If you choose “no”, your identity will remain anonymous but your review may still be made public.

**Do you want your identity to be public for this peer review?** For information about this choice, including consent withdrawal, please see our [Privacy Policy](https://www.plos.org/privacy-policy).

Reviewer #1: No

Reviewer #2: No

[NOTE: If reviewer comments were submitted as an attachment file, they will be attached to this email and accessible via the submission site. Please log into your account, locate the manuscript record, and check for the action link "View Attachments". If this link does not appear, there are no attachment files.]

While revising your submission, please upload your figure files to the Preflight Analysis and Conversion Engine (PACE) digital diagnostic tool, <https://pacev2.apexcovantage.com/>. PACE helps ensure that figures meet PLOS requirements. To use PACE, you must first register as a user. Registration is free. Then, login and navigate to the UPLOAD tab, where you will find detailed instructions on how to use the tool. If you encounter any issues or have any questions when using PACE, please email PLOS at [figures@plos.org](mailto:figures@plos.org). Please note that Supporting Information files do not need this step.
