# Supplementary material for: Awareness of diagnosis, treatment plan and prognosis among patients attending public hospitals and health centers in Addis Ababa, Ethiopia
Source: PLoS One. 2022 Jun 23;17(6):e0270397. doi: 10.1371/journal.pone.0270397 (PMC9225456; doi:10.1371/journal.pone.0270397)
Supplement: Attachment — Submitted filename: Responses to reviewers_2.docx [file pone.0270397.s002.docx]

**Responses to reviewers’ comments**

1-Decription of prognosis assessment in the African setting is important. More information how this was done would be helpful. For example, in the method section specifically described what questions were used and how was prognosis knowledge defined. Specifically, was it based on threat to life in short or long term, or possibility of disease chronicity? How were diagnosis in Ethiopian same or different to conditions in the reference's articles: cancer, schizophrenia in Western medicine setting? Explicit description of who with what diagnosis met prognosis understanding criteria would help.

**Response**:

Prognosis in this study was determined based on threat to life in short or long term and need for continued treatment (chronicity). Patients were asked if their doctors have told them about possibility for cure (if they were told whether the treatment will cure them or if they needed to take it for life), as well as life expectancy (threat to life). What the patients reported was compared with the patient record and/or report from the treating physician. Any patient with any condition who has been told that he will be cured after the treatment, any patient who has a knowledge of the chronicity of the disease and any patient who knows the estimated number of months/years s/he is left with, is categorized as “know their prognosis” when verified by the document/report from physician and the panel of physicians. A revision is included in the manuscript.

2-The multiple regression analysis could use further clarity and description in the method section and table 3 (the title does not state that this is a multiple regression. Also, it is not clear what are separate models, what are controlling variables, also interactions assessment is not addressed, the issue of multiple analysis alpha level adjustment mentioned by the editor also needs attention).

**Response**: We have refined the data analysis section based on your comments. The title of table 3 now indicates that the outputs are from multiple logistic regression. By separate models, we mean we ran three models- one for awareness of diagnosis as an outcome, a second for awareness of treatment, and a third for awareness of prognosis as outcome. As the multiple logistic regression analysis was exploratory, the OR for an independent variable were controlled for all other independent variables in the model. We have checked interactions among key variables in model 3. But we didn’t get any significant interaction. We appreciate the comment on alpha level adjustment. However, we think this would increase type II error.
